# Bringing women’s voices to PMTCT CARE: adapting CARE’s Community Score Card© to engage women living with HIV to build quality health systems in Malawi

**DOI:** 10.1186/s12913-020-05538-2

**Published:** 2020-07-22

**Authors:** Anne Laterra, Tegan Callahan, Thumbiko Msiska, Godfrey Woelk, Pari Chowdhary, Sara Gullo, Patience Mgoli Mwale, Surbhi Modi, Felluna Chauwa, Dumbani Kayira, Thokozani Kalua, Etobssie Wako

**Affiliations:** 1 CARE USA, 151 Ellis Street NE, Atlanta, GA USA; 2 Centers for Disease Control and Prevention, Atlanta, GA USA; 3 CARE Malawi, Lilongwe, Malawi; 4 Elizabeth Glaser Pediatric AIDS Foundation, Washington, D.C USA; 5 Independent consultant, Atlanta, GA USA; 6 Elizabeth Glaser Pediatric AIDS Foundation, Lilongwe, Malawi; 7 Centers for Disease Control and Prevention, Lilongwe, Malawi; 8 Ministry of Health, Lilongwe, Malawi

**Keywords:** PMTCT, HIV, Quality improvement, Social accountability, Participation, Voice, User involvement, Patient involvement, Patient engagement

## Abstract

**Background:**

Coverage of prevention of mother-to-child transmission of HIV (PMTCT) services has expanded rapidly but approaches to ensure service delivery is patient-centered have not always kept pace. To better understand how the inclusion of women living with HIV in a collective, quality improvement process could address persistent gaps, we adapted a social accountability approach, CARE’s Community Score Card© (CSC), to the PMTCT context. The CSC process generates perception-based score cards and facilitates regular quality improvement dialogues between service users and service providers.

**Methods:**

Fifteen indicators were generated by PMTCT service users and providers as part of the CSC process. These indicators were scored by each population during three sequential cycles of the CSC process which culminates in a sharing of scores in a collective meeting followed by action planning. We aggregated these scores across facilities and analyzed the differences in first and last scorings to understand perceived improvements over the course of the project (z-test comparing the significance of two proportions; one-tailed *p*-value ≤ .05). Data were collected over 12 months from September 2017 to August 2018.

**Results:**

Fourteen of the fifteen indicators improved over the course of this project, with eight showing statistically significant improvement. Out of the indicators that showed statistically significant improvement, the majority fell within the control of local communities, local health facilities, or service providers (7 out of 8) and were related to patient or user experience and support from families and community members (6 out of 8). From first to last cycle, scores from service users’ and service providers’ perspectives converged. At the first scoring cycle, four indicators exhibited statistically significant differences (*p*-value ≤ .05) between service users and service providers. At the final cycle there were no statistically significant differences between the scores of these two groups.

**Conclusions:**

By creating an opportunity for mothers living with HIV, health service providers, communities, and local government officials to jointly identify issues and implement solutions, the CSC contributed to improvements in the perceived quality of PMTCT services. The success of this model highlights the feasibility and importance of involving people living with HIV in quality improvement and assurance efforts.

**Trial registration:**

Trial registration: ClincalTrials.gov NCT04372667 retrospectively registered on May 1st 2020.

## Background

In 2011, the global community joined together under the Global Plan Towards the Elimination of New HIV Infections Among Children and Keeping Mothers Alive to set the course for global elimination of vertical transmission of HIV [1]. As a result, prevention of mother-to-child HIV transmission (PMTCT) services in Global Plan countries rapidly expanded, and the mother-to-child transmission rate fell from 22.4% in 2009 to 8.9% in 2015 [2]. These successes were due in large part to the impressive expansion of lifelong antiretroviral treatment (ART) to pregnant and breastfeeding mothers living with HIV [3]. Building on these successes, global stakeholders came together again, this time calling for an end to the AIDS epidemic by 2030 [4] and revised treatment guidelines to include livelong ART for all people living with HIV -- the Treat All approach [5]. Despite these commitments and dramatic increases in service coverage, gaps in retention in PMTCT care and timely testing of HIV-exposed infants remain [6, 7]. In Malawi, recent program data suggests rapid improvement over the past few years in HIV-exposed infant testing at 6 weeks. Current data from sites supported under the US Presidents Emergency Plan for AIDS Relief suggests coverage of testing at 6 weeks is around 75% [8], up considerably from previous studies estimating that less than half of all HIV-exposed infants completed the recommended nucleic acid-based test within the first 6 weeks [9, 10] . Despite these improvements, coverage and data quality is variable at the sub-national level. In addition, studies in Malawi have shown women who initiate ART through antenatal care (ANC) have persistently higher loss-to-follow-up rates compared to those who begin treatment through general HIV services (i.e. not when pregnant) [11].

These persistent challenges highlight the need for new strategies that strengthen the quality of PMTCT care and meaningfully engage mothers living with HIV in improving the quality of their own treatment and the care of their infants. Multiple studies have found that the quality, particularly the *perceived* quality, of health services is important and meaningfully affects health-seeking behavior including decisions to initiate care, remain in care, and adhere to treatment guidelines [12–15]. It is critical to address this shortcoming within PMTCT programming because quality of services is particularly essential to the effective management, treatment and prevention of chronic and complex conditions like HIV [16]. Indeed, two dimensions that render populations and individuals at increased risk for poor quality health care services are the continued stigmatization of certain conditions, including HIV and AIDS, and demographic vulnerabilities such as gender identity, both of which are particularly applicable to mothers living with HIV [16].

Despite this growing recognition that quality of care, not just coverage, is integral to improved PMTCT outcomes [16, 17], quality assurance and improvement efforts have often not kept up with the dramatic pace of expanded PMTCT service coverage [18]. Where quality improvement efforts are well resourced, they often focus almost exclusively on staff training and capacity building, service integration, information management systems and reminders, and laboratory support [19, 20]. These efforts do little to engage HIV-positive women in their own care, involve them in quality improvement and accountability processes, or understand their lived experience with treatment [19–21]. As a result, they often fall short of making significant improvements in the user experience [21–23].

In order to better understand how the inclusion of women living with HIV in a collective, quality improvement process could address persistent retention and adherence gaps, we adapted a social accountability approach, CARE’s Community Score Card© (CSC), to the PMTCT service delivery context. This paper presents select results of a mixed-methods, pre / post evaluation: the key issues that service users and providers identified as necessary to accelerate PMTCT outcomes and an analysis of perceived progress in these areas over the course of the CSC process. We also offer reflections on the successes and limitations of the adapted approach. Analyses of changes in clinical outcomes and participants’ experiences with the approach are presented elsewhere [24].

## Methodology

### Intervention description

**Fig. 1:**
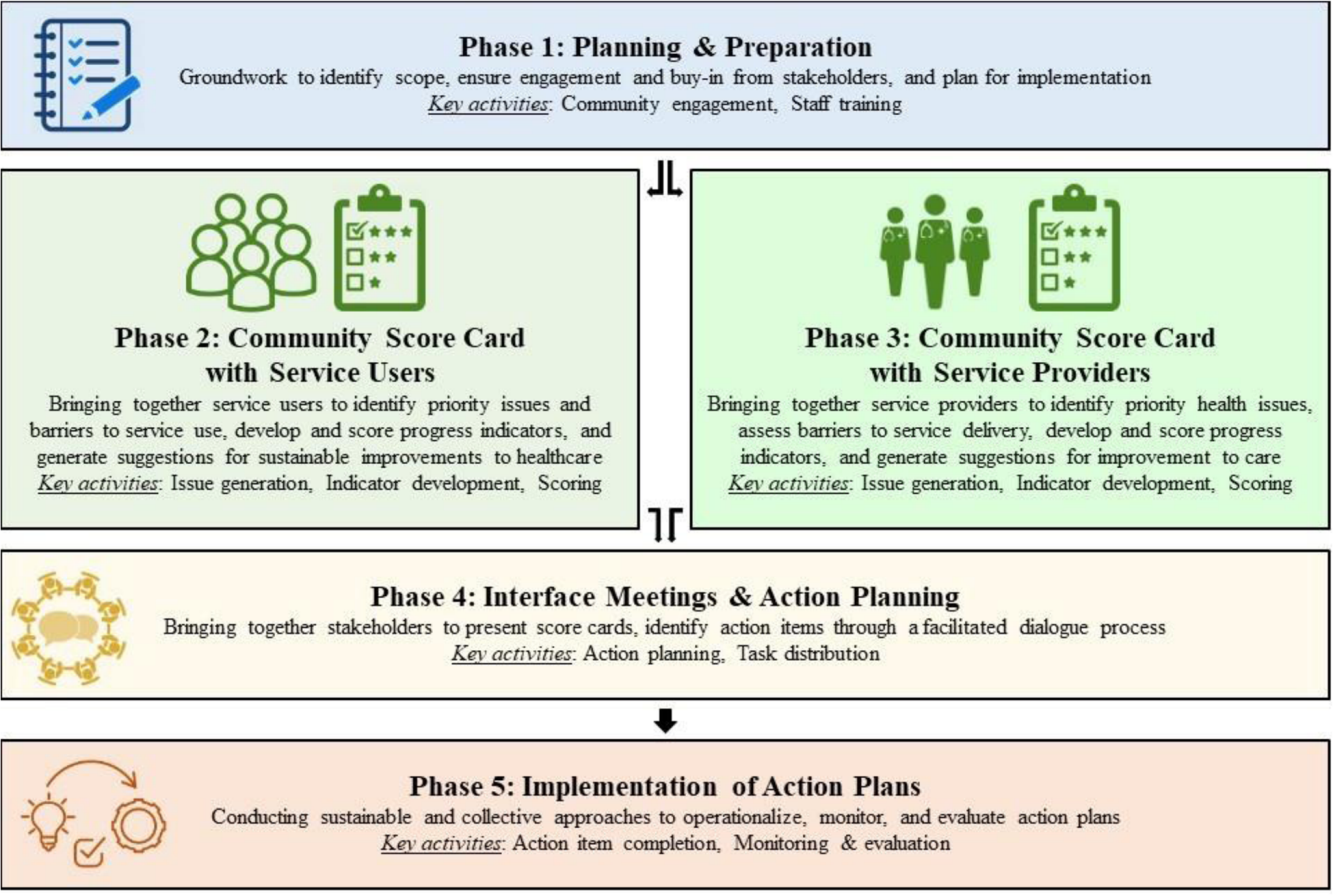
CARE’s Community Score Card Process

To address these challenges, an inclusive quality improvement approach that empowers mothers living with HIV and their providers to systematically identify and overcome barriers to quality of care and identify joint solutions is needed. CARE’s Community Score Card©(CSC), a community-engagement approach that brings together service users and service providers at the local level to collectively share feedback and improve the quality of services, could potentially serve as one such approach. CARE’s CSC is grounded in the principles of social accountability, the belief that the mechanisms which allow citizens themselves to engage directly with duty bearers increase public officials’ accountability to their commitments and responsibilities, and in those of patient-centered care, a respect for a patient’s preferences, needs and values and a commitment to provide responsive, consultative care [25, 26]. The approach has been shown to improve service use and access, satisfaction with services, and accountability to patients’ needs and desires [27, 28]. CARE’s CSC approach is described more extensively elsewhere [27, 28]; however, briefly, the CSC consists of five-phases of implementation (see Fig. 1). Each of the five phases makes up a single cycle of the CSC process. Essential to the success of the CSC is the fact that these cycles are repeated on a regular basis, facilitating an ongoing quality improvement process and not a one-off event or activity. Progress on issues identified by those engaged in the process is assessed using score cards that track context-specific indicators, and action plans that document collective action to which participants in the process commit.

While CARE’s CSC has demonstrated positive impact on health services related to general maternal, neonatal and child health, and other health service domains [27, 28], at the time of the launch of this project it had not been adapted, implemented, or evaluated in a HIV health service delivery setting. As HIV remains a stigmatized health issue [29–31], people living with HIV too often are not given opportunities to voice their specific concerns and needs, and lack trust and confidence in the health system’s ability to provide confidential, tailored, and respectful care as a result [32, 33].

To address this gap, CARE partnered with the Malawi Ministry of Health through the support of the Elizabeth Glaser Pediatric AIDS Foundation (EGPAF) and the US Centers for Disease Control and Prevention to adapt and implement the CSC approach with PMTCT service providers and with mothers who use PMTCT services. Key considerations within this adaptation included operationalizing the approach through the clinic platform and 24-month PMTCT treatment cycle; alignment with the national HIV control strategy; and privacy of mothers living with HIV. Specific adaptations within each phase are described below.

#### Phase 1 planning and preparation

Typically, this phase includes the groundwork to identify scope, ensure buy-in from community and other stakeholders, and plan for implementation. Unlike previous iterations of CARE’s CSC that worked with communities directly to engage service users in the project, this adaptation relied on clinics to serve as the platform for both recruitment and implementation of the approach. Facilities with high maternal HIV-positive patient loads and poor PMTCT indicators were specifically targeted for implementation. Mothers initiating ART for PMTCT following ANC visits were invited to participate in the process. HIV-positive breastfeeding mothers were also identified and invited through outreach to existing support groups and via mother-infant-pair clinic days.

Typically, the CSC trains community members to serve as co-facilitators in issue generation, scoring, and interface meetings at each site. For the PMTCT adaptation, expert clients^1^ and mentor mothers^2^ were prioritized as facilitators and trained to play this role, serving as a bridge between HIV-positive community members and the health system.

#### Phase 2 conducting the CSC with service users

In this stage, implementers bring together service users to identify priority issues, list and score indicators to measure progress, and generate suggestions for sustainable improvements. To promote a focus on PMTCT-specific issues, the adapted CSC restricted participation to PMTCT service users (HIV-positive pregnant and lactating mothers). As opposed to CARE’s previous CSC implementation experiences that were held publicly to promote accountability and broad-based collective action, the PMTCT CSC meetings were held at secluded off-site locations. The issue generation process also employed a PMTCT-specific discussion guide, created to surface issues specific to initiation and retention in treatment and uptake of early infant diagnosis services. The assumption was that by engaging only mothers who had used PMTCT services, and focusing discussions around barriers to these services specifically, the action plans and solutions identified through the CSC process would creatively address the barriers specific to PMTCT.

#### Phase 3 conducting the CSC with service providers

Phase 3 is similar to phase 2 but focuses on service providers rather than service users. This phase allows service providers to share their own perspectives on the successes and challenges of delivering health services. Unlike in primary health centers where all clinic staff participated in issue generation and scoring exercises, in district hospitals participation was restricted to staff representatives from departments that specifically served HIV-positive pregnant and breastfeeding mothers: doctors, clinical officers, nurses and midwives from ANC and HIV/ ART clinical departments.

#### Phase 4 Interface meetings and action planning

To promote collective action and accountability, interface meetings convene not just service users and service providers but members of health advisory committees, district health management teams, and other relevant stakeholders to discuss the score cards and develop a joint action plan. Since interface meetings include these various groups, the adapted CSC for PMTCT included an option for HIV-positive participants to elect a representative to present their scores on their behalf, sometimes an expert client or mentor mother.

#### Phase 5 implementation and monitoring of action plans

To more rapidly address challenges of prevention of transmission to infants over a defined risk period, the implementation period for action plans under the adapted CSC was abbreviated from the typical 6 months to 3 months.

### Study setting and site selection

The study was conducted at 11 health facilities across Dedza and Ntcheu districts, including nine health centers (primary care level) and two district hospitals (secondary level). Dedza and Ntcheu districts are in Malawi’s central region. The nine health centers were located in small towns or rural parts of the District whereas the two district hospitals were located in the more urban and peri-urban areas. Sites were selected purposively through review of routine PMTCT program monitoring data. Selected facilities met the following criteria: sufficient volume of newly identified HIV-positive pregnant mothers each year (minimum of 25); less than an 85% 6-month ART retention rate among mothers; and 6-week infant diagnosis performance that fell below the national average.

### Recruitment

#### Service user recruitment

Recruitment of PMTCT service users for participation in the CSC process occurred through two distinct avenues: support group-based recruitment and clinic-based recruitment. Clinic-based recruitment, conducted by clinic-staff, offered the most efficient way to recruit newly diagnosed HIV-positive mothers at the onset of their PMTCT journey. This recruitment was supplemented with a parallel recruitment exercise, conducted by project staff, among support group members as a way to include women who were already diagnosed and so would not be captured in the clinic-based recruitment process. Identifying, mapping and connecting with support groups for people living with HIV was part of the first phase of the adapted PMTCT CSC. Through these groups, pregnant and breastfeeding mothers living with HIV were invited to participate in the PMTCT CSC process.

Newly diagnosed HIV-positive mothers and women who may not have yet joined a support group, were identified and recruited from the health facilities while accessing clinical services. Independent of the recruitment pathway, mothers who were interested in participating received a detailed written description of the process and consented to participate using a signature or thumbprint. A total of 822 mothers were recruited to participate.

#### Service provider recruitment

Service providers were recruited to participate in the CSC intervention in close collaboration with facility managers and the District Health Management Teams (DHMT). The project was introduced to the DHMT and then to in-charges and staff at each facility through in-person meetings and health workers were invited, at this time, to participate. Because a wide breadth of providers play a role in how women access and utilize PMTCT services, the program aimed to identify service providers from every level of service delivery in the primary health care facilities. In the two District Hospitals, representatives from ANC and ART clinics specifically participated due to operational limitations of including all clinic staff. Once health workers were invited and consented to participate, they were oriented on the importance of maintaining confidentiality during the CSC as part of the initial meetings and project start-up. A total of 64 health workers provided written consent.

#### Stakeholder recruitment

Select leaders and stakeholders from the broader community were engaged in the interface meetings and action planning phases of the CSC process. Engagement of these stakeholders increased accountability on issues identified through earlier phases of PMTCT CSC implementation. These leaders were identified through introductory meetings prior to the start of the project, and throughout its execution as different challenges and solutions emerged. These included religious leaders, DHMT members, leaders of governance structures (i.e., health advisory committees, village development committees, and village health committees), village chiefs, traditional authorities, and politicians (members of Parliament and councilors).

### Participation

PMTCT service users and providers were identified through the recruitment strategies described above and invited to attend each stage of the PMTCT CSC process. This included an initial issue generation meeting during the first cycle and then subsequent scoring, and interface meetings in all three cycles. To ensure that both issues and solutions addressed challenges unique to each facility, all meetings were facility specific, meaning they involved only service users and service providers associated with that particular facility. Each cycle took about 1 month to complete across all 11 health facilities, followed by a three-month action plan implementation and monitoring period. Together, all three cycles were conducted over a period of 12 months, from September 2017 to August 2018.

### Participatory development of indicators

Score card indicators were developed in a consultative, participatory process based on the issues identified during the issue generation meetings. Once issues were identified, an indicator development meeting was held. During these meetings, facilitators listed, reviewed, and discussed the priority recommendations that came out of the issue generation process. Major themes were classified into distinct domains and a perception-based indicator was created. For example, issues such as reluctance of male partners to get tested and low participation of male partners in ANC visits and decisions around infant care and testing; were classified into an indicator of “Level of male involvement on PMTCT Issues”.

Once indicators were created, service users and service providers met separately across each of the 11 facilities to conduct scoring meetings. During scoring meetings, participants discussed each indicator and agreed on a perception-based score using a scale of 0 to 100. This process generated two separate score cards per health facility – one from the service users’ perspective and a second from the service providers’ perspective. These two score cards were presented and discussed during the Interface Meetings and informed the development of subsequent action plans. The same indicators were used across all 11 intervention sites (see Table 1).

**Table 1:** PMTCT CSC Indicators by locus of control and component of high-quality health system

PMTCT^a^ CSC Indicator	Locus of control^b^	Component of High-Quality Health System^c^
Attitude and commitment of PMTCT service providers	Provider	Positive user experience
Disclosure support and maintenance of confidentiality of HIV^a^ positive status	Provider	Positive user experience
Prevalence of stigma and discriminatory behaviors towards women living with HIV	Provider	Positive user experience
Level of male involvement on PMTCT Issues	Community	Population
Availability of adequate infrastructure, equipment and supplies to deliver PMTCT services	Health System	Tools
Availability of trained health workers capable of providing PMTCT services	Health system	Workforce
Adherence to clinical advice and ART^a^ treatment by service user	Individual / Patient	Confidence in systems
Access to high quality PMTCT counseling and information	Health System	Competent care and systems
Accessibility to facilities providing PMTCT services	Healthy System	Platforms
Influence of cultural and religious beliefs on access to and utilization of HIV testing and treatment services	Community	Population
Availability of social support from leaders, community-based organizations and relatives at community level	Community	Population
Convenient and timely access to HTC^1^/ART/PMTCT/EID^a^ services and results at facility level	Health Facility	Competent care and systems
Availability of integrated services	Health System	Competent care and systems
Follow-up of defaulters	Health System	Competent care and systems
Level of supervisory support	Health System	Tools

^a^ PMTCT: prevention of mother-to-child transmission; HIV: human immunodeficiency virus; ART: antiretroviral therapy; HTC: HIV testing and counseling; EID: early infant diagnosis

^b^ Locus of control was assigned as either individual / patient, provider, health facility, community or health system based on where / who had the highest capacity and authority to effect change in the indicator

^c^ Component was assigned based on Kruk et al.’s high quality health system framework components

### Data Collection

During these meetings, data in the form of scores for each indicator were recorded using a paper template, posted on large poster paper so that all participants could view and confirm scores were recorded accurately. Upon completion of each round, scores for all 11 facilities were entered by CARE project staff into an Excel database. Quality assurance was conducted by the Technical Advisor supporting this work who examined the database after each month, identified any missing values or values outside of the plausible range, and through an audit of the paper records, made any necessary corrections.

### Data analysis

For our analysis we categorized the PMTCT CSC Indicators based on locus of control and by Kruk et al.’s domains and components of a high-quality health systems [16]. Locus of control was assigned across one of six categories as either individual or patient, provider, health facility, community, or health system based on where or who had the highest capacity and authority to effect change in the indicator. For example, availability of trained health workers was categorized as within the health system locus of control because, in this context, human resource allocations and training decisions are primarily made at the district level in accordance with national-level guidance and resourcing. Each indicator was also categorized based on one of the ten components of high-quality health systems defined by Kruk et al. [16].

We examined changes in scores aggregated across service provider and service user populations from first and last cycle. While scores were collected at individual sites, for this analysis we averaged the scores for the first and last cycle across the 11 sites to arrive at three distinct scores per time period, per indicator: a service user score and a service provider score, and a combined score that averaged across both service users’ and service providers’ perspectives. The analysis below presents the percent change from first to last cycle of the combined scores aggregated across all sites and across both scoring populations (service users and providers), rounded to the nearest whole number. We also compare the absolute percentage point difference between service user and service provider scores, aggregated across all sites, at first and last cycle. Differences were assessed using a Z test and *p*-values ≤ .05 were considered statistically significant. Analysis was conducted using Microsoft Excel [34].

## Results

### Issue generation and indicator development

Fifteen indicators (Table 1) were identified through the PMTCT CSC. The indicators cut across all loci of control with most falling within the health system’s [7], providers’ [3], and community’s [3] control. In terms of quality, these indicators represented a more limited number of components. Of the ten components of high-quality health systems, seven were represented here. These include competent care and systems [4], population [4], positive user experience [2], tools [2], confidence in system [1], workforce [1], and platforms [1].

### Score card results

**Fig. 2:**
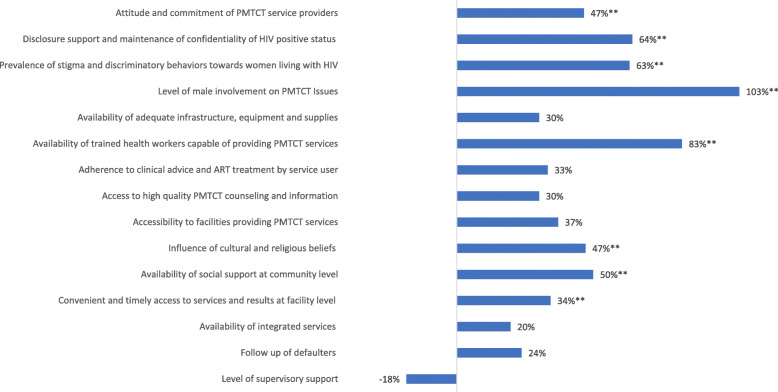
Percent Change in Score Card Indicators from First to Final Scoring; z-test comparing the significance of 2 proportions (one-tailed *p*-value).; ** *p*-value ≤ .05

Indicator scores aggregated across all 11 sites and both populations (service user and provider) increased by an average of 43% from the first to last round of the PMTCT CSC process. Only one indicator, level and quality of supervisory support, had a lower score during the final cycle compared to the first cycle, a 18% decrease (from 76 to 62) although this change was not statistically significant (*p = .76)*. The highest increase in indicator score from the first to last cycle was seen in male involvement in PMTCT issues (103% increase; from 29 to 59 points), and availability of health workers (83% increase; from 40 to 73 points) (Fig. 2).

Six other indicators experienced statistically significant improvements including more positive attitude and commitment of service providers (47%; from 58 to 85 points), increased disclosure support and better maintenance of confidentiality (64%; from 42 to 69 points), reduced prevalence of stigma and discriminatory behaviors (63%; from 49 to 80 points), more positive influence of cultural and religious beliefs on access to and utilization of services (47%; from 53 to 78 points), increased availability of social support at community level (50%; from 52 to 78 points), and more convenient and timely access to services (34%; from 64 to 86 points).

#### Score card results by locus of control

**Fig. 3:**
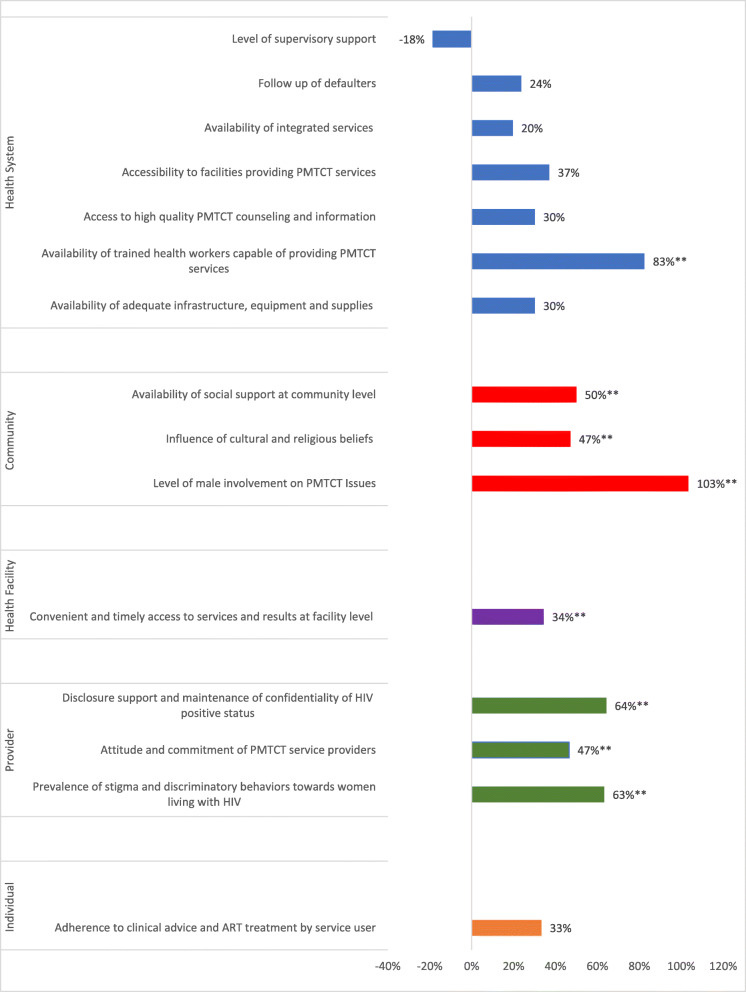
Percent Change in Score Card Indicators from First to Final Scoring by Loci of Control; z-test comparing the significant of 2 proportions (one-tailed p-value). ** *p*-value ≤ .05

Figure 3 illustrates changes in score card indicators by locus of control (Fig. 3). All indicators within the provider’s, health facility’s and community’s loci of control experienced statistically significant improvements from first to last cycle. Conversely, most indicators falling at either end of the spectrum, either individual’s or health system’s locus of control, did not improve significantly over the course of the project. The one exception to this is the indicator *availability of trained health workers*, which falls within the health system’s locus of control as it concerns human resource management decisions made at national, zonal, and district level. This indicator saw a statistically significant improvement.

#### Score card results by domain and component of high-quality health systems

**Fig. 4:**
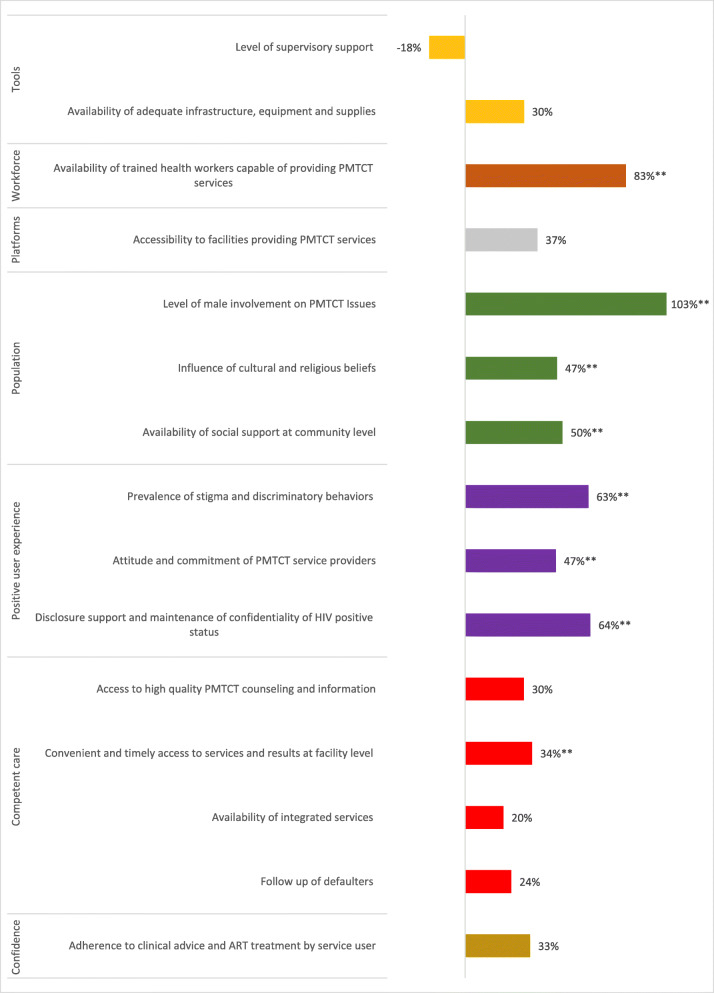
Percent Change in Score Card Indicators from First to Final Scoring by component of high-quality health system; z-test comparing the significant of 2 proportions (one-tailed p-value). ** *p*-value ≤ .05

All indicators within the population (such as community and family involvement and supportive social norms) and positive user experience components of care experienced statistically significant improvements. In addition, availability of trained health workers, in the workforce component, and access to services and results at the facility level, within the competent care component, also experienced statistically significant increases (Fig. 4).

#### Difference in service user and service providers score card results

**Fig. 5:**
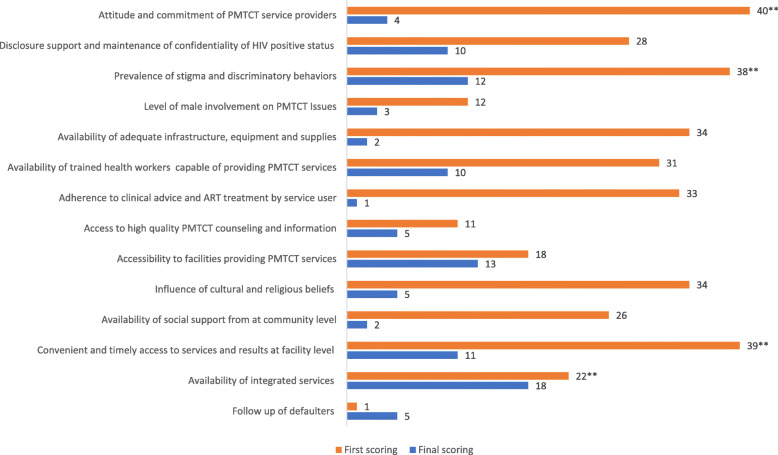
Absolute percentage point difference between service user and service provider scores at first and final scoring; z-test comparing the significant of 2 proportions (one-tailed p-value). ** *p*-value ≤ .05

At first scoring, the average service user score was lower than the average service provider score for 13 of the 14 jointly scored indicators (note: level supervisory support indicator was scored only by service providers and so is not included in this analysis). The difference was statistically significant for four indicators: attitude and commitment of PMTCT service providers, prevalence of stigma and discriminatory behaviors, convenient and timely access to services and results, and availability of integrated services. At the final scoring there were no statistically significant differences between service user and provider scores. This convergence appears to be largely due to improvements in the service user’s scores. Indicators that experienced the highest reduction in the difference between the two scores, were attitude and commitment of PMTCT service providers, adherence to clinical advice and ART treatment, and availability of adequate infrastructure (Fig. 5).

## Discussion

This is the first attempt to adapt and assess CARE’s CSC approach to quality improvement efforts in the PMTCT service delivery context. We found that by engaging PMTCT service users, a broad set of indicators reflecting quality of care and wider systems- and community-level supports and barriers were identified. The indicators that surfaced as a result of this process echo previous studies which have identified barriers experienced by PMTCT service users, including issues of stigma and discrimination [35, 36], the need for support in disclosing to partners [36], the value of community support [21, 37], the importance of consistently available supplies, and the desire for more integrated care [21, 38].

In examining the change in indicators, we found that all but one increased over the course of the PMTCT CSC process, with eight indicators showing statistically significant increases. All but one of these eight indicators, availability of trained health workers, fell within either the provider’s, health facility’s or community’s locus of control. This is similar to previous CSC and social accountability experiences which found that unlocking resources and affecting change is easiest and most feasible, in the short and medium term, around issues and solutions that are within the immediate control of providers and, to a lesser extent, facilities and community members [27, 28, 39]. This may have been particularly true with the PMTCT CSC adaptation due to the shortened action periods. Systems-level change likely takes longer to realize and may require additional efforts to strengthen the health sector’s capacity to respond to demands for accountability [39].

The significant increase in the perceived availability of trained PMTCT health workers is unique in that it was the only indicator within the health system’s locus of control that experienced a statistically significant improvement. This can potentially be explained by the engagement of the DHMT in the interface meetings where they committed to the deployment of additional human resources. Communities also collectively mobilized to repair or build staff accommodations and facilities, a requirement for deployment and retention of health care workers in these settings but an action that is more squarely in the control of community members themselves but has an impact on health system-level human resourcing.

In addition to positive changes in indicators, this analysis aims to understand what components of quality health systems were best addressed through the PMTCT CSC process. We found that the CSC process was most effective at tackling issues related to the user experience (respect, autonomy, confidentiality, choice, patient voice and values) and the population (individuals, families, communities and norms as integral to better health outcomes) components of quality. All indicators concerning user experience increased significantly from first to final cycle.

In addition to exploring improvements in indicator scores aggregated across users and providers, we also explored how agreement, or disagreement, between service users and service providers changed over the three cycles. Our results illustrate that, across the board, scores from each perspective began to more closely approximate each other over the life of the project. Increasing agreement on scores is an important indication of an effective CSC process. The approach aims to foster a shared understanding and appreciation of strengths and weaknesses as a first step towards identifying successful and meaningful collective and collaborative action. As Armstrong et al. asserts, clear roles for service user engagement in quality improvement efforts are needed. One crucial role service users can, and do, play in this process is that of “patients as persuaders”—persuading service providers and other stakeholders of the importance of existing issues or barriers and influencing how these should be addressed [23]. Our results suggest that service users engaged in the PMTCT CSC process may have served this function, drawing attention to previously neglected components of quality of care that required collective solutions and consensus building among key stakeholders.

A review of the monitoring and action plans set forth in the interface meeting and assessed in subsequent follow-up meetings illustrated additional resources and solutions that were unlocked as a result of this process. We found that interface meetings served as a platform for women to express issues related to other services, such as outpatient care, maternity, and family planning. This illustrates that women view and experience PMTCT services as integrated within their broader health care experience. This meant that the PMTCT CSC not only tackled PMTCT-specific issues but also worked to mobilize facility- or community-wide resources. Ensuring that HIV-positive women have a voice in quality improvement efforts can yield positive results in quality of care, building trust and accountability between service users and service providers, and generate solutions with wider-reaching implications.

### Lessons learned

Over the course of implementation of the adapted CSC model the team learned and refined which key stakeholders were most crucial to include in the process. Two distinct learnings surfaced from this experience. First, religious leaders emerged as extremely committed and energized champions. Pastors Fraternal organizations in each District played a key role coordinating with the DHMT, mobilizing wider community support, and combating harmful religious messages concerning HIV treatment. This is a promising finding given the global HIV response’s long-standing commitment to partnerships with faith-based organizations [40] and could signal a meaningful role for these organizations in future accountability processes. Second, while women were encouraged to invite male partners at their own discretion, to avoid any breaches of confidentiality or disclosure, more robust male engagement could likely have been achieved by involving interested couples together from the start of the process and providing more disclosure support as part of that approach.

### Limitations

The primary limitation of this analysis and findings is the nature of the indicators themselves. In keeping with the participatory, locally-led nature of the CSC approach, these indicators are qualitative and perception-based. As such, scores may vary for reasons that are not related to actual health system performance or quality of care, such as changes in individuals who participated in the scoring process and differences in the facilitation between groups and over time. Aggregating scores across facilities and between service users and service providers may also mask participant group-and facility-level variability which, in some cases, was substantial. Despite these limitations, these scores are valid as indicative measures of the general status of key issues identified as most meaningful to service users and providers and their participant-driven nature aligns with the principles of patient-centered care and social accountability approaches that focus on the lived and perceived experiences of clients. As such, they can be analyzed to understand generalized changes in perceptions of improvement over time and across sites. Future research on how perceptions of quality compare to objective measures and the relative importance of each would contribute to deeper understanding.

While we did see improvement in these perception-based indicators and feel confident that these represent general improvements over the course of the implementation period, in this analysis we did not intend to examine the relationship between improvement in these elements and clinical outcomes. There is, however, substantial existing literature that illustrates that improving the quality of care, particularly patient-centered elements of quality, and increasing patient engagement improves health outcomes particularly in chronic conditions [41, 42]. Equally important, is the principle of quality care as a human right, valued independently of subsequent improvements in clinical outcomes [26].

We also did not see statistically significant improvement in all indicators suggesting the PMTCT CSC specific actions was not effective at achieving positive changes across all loci of control or domains of quality. This may be due to the shortened periods between cycles within this adaptation, from 6 months to 3 months, and the relatively short implementation period overall. These two factors may have made it more difficult to achieve statistically significant improvements among the indicators that fell within the health system’s locus of control, as these indicators perhaps require additional time and sustained effort to see improvements. We suggest that future research explores if and how these types of approaches, if implemented over a longer period of time, overcome systemic barriers to quality care.

Finally, because we did not have a comparison, or control group, we cannot be certain that changes in perceived quality illustrated through the PMTCT CSC process were not caused by other, external factors unrelated to the implementation of the intervention. We do, however, have qualitative data that both health care workers and service users provided as part of the scoring process to indicate why and how scores changed from round to round, and much of the justification for these changes (or lack of changes) relates directly to actions taken (or not taken) as a result of the PMTCT CSC process. We feel these data give us reasonable confidence in the contribution of the PMTCT CSC to the improvements presented here.

## Conclusions

Throughout the implementation of the PMTCT CSC, we observed improvement in 14 of the 15 indicator scores, with statistically significant increases in eight, between the first and third cycles of the CSC process. In general, indicators that fell within the providers’, health facilities’, or communities’ control and those that addressed the user experience and population components of a quality health system, such as community and family involvement and supportive social norms, were most likely to experience substantial improvements. Providers’ and service users’ scores for almost all indicators were closely aligned at final scoring. Our findings demonstrate that engagement methodologies can be successfully used to increase perceived quality within targeted health services such as PMTCT. Our findings suggest that delivery of quality HIV services in lower-resource settings, such as Malawi, will be strengthened and supported by approaches that bring service users’ voices and lived experience to bear in quality improvement processes. This adapted PMTCT CSC approach offers one such method by allowing service users to identify priorities and concerns and offering repeated opportunities for collective solution generation and action.

## Supplementary information

**Additional file 1.**

## Data Availability

All data analyzed for this manuscript are included in this published article [see Additional file 1].

## Abbreviations

ART: Antiretroviral therapy
ANC: Antenatal care
CSC: Community Score Card
DEC: District Executive Committee
DHMT: District Health Management Team
EGPAF: Elizabeth Glaser Pediatric AIDS Foundation
HIV: Human Immunodeficiency Virus
PEPFAR: President’s Emergency Plan for AIDS Relief
PMTCT: Prevention of Mother-To-Child Transmission

## Footnotes

1 HIV-positive individuals trained and deployed to assist salaried health care workers in community-based provision of services

2 HIV-positive women working in local communities and health facilities to ensure women living with HIV are supported and linked with correct clinical services.
